# Inter-rater agreement of CDC criteria and ASEPSIS score in assessing surgical site infections after cesarean section: a prospective observational study

**DOI:** 10.3389/fsurg.2023.1123193

**Published:** 2023-08-22

**Authors:** Giovanni Delli Carpini, Luca Giannella, Jacopo Di Giuseppe, Marco Fioretti, Ilaria Franconi, Ludovica Gatti, Keti Sabbatini, Michele Montanari, Chiara Marconi, Elisa Tafuri, Luisa Tibaldi, Mariasole Fichera, Davide Pizzagalli, Andrea Ciavattini

**Affiliations:** Obstetrics and Gynecologic Section, Department of Odontostomatological and Specialized Clinical Sciences, Università Politecnica delle Marche, Ancona, Italy

**Keywords:** surgical site infections, cesarean section, cesarean delivery, CDC criteria, ASEPSIS score, inter-rater agreement, surveillance, reliability

## Abstract

**Objective:**

To assess and compare the inter-rater agreement of the CDC criteria and the ASEPSIS score in identifying surgical site infections after cesarean section.

**Methods:**

Prospective observational study including 110 patients subjected to a cesarean section at our institution. Surgical wounds were managed according to standard care and were photographed on the third, seventh, and thirtieth postoperative day or during any evaluation in case of complications. Three expert surgeons reviewed the prospectively gathered data and photographs and classified each wound using CDC criteria and the ASEPSIS score. The inter-rater agreements of CDC criteria and ASEPSIS score were determined with Krippendorff's Alpha with linear weights and compared with a confidence interval approach.

**Results:**

The weighted *α* coefficient for CDC criteria was 0.587 (95%CI, 0.411–0.763, *p* < 0.001, “moderate” agreement according to Altman's interpretation of weighted agreement coefficient), while the weighted *α* coefficient for the ASEPSIS score was 0.856 (95%CI, 0.733–0.980, *p* < 0.001, “very good” agreement).

**Conclusion:**

ASEPSIS score presents a “very good” inter-rater agreement for surgical site infections identification after cesarean, resulting in a more objective method than CDC criteria (“moderate” inter-rater agreement). ASEPSIS score could represent an objective tool for managing and monitoring surgical site infections after cesarean section, also by photographic evaluation.

## 1. Introduction

Cesarean Section (CS) is the most frequent obstetric procedure performed all over the world, with a global rate of 18.6% of all births (range 6.0%–27.2%), steadily increasing in the past decade (1). CS is associated with high healthcare costs and an intrinsic risk of short-term and long-term complications, including surgical site infections (SSIs) (2). After CS, the incidence of SSIs varies between 4.9% and 9.8% (3), and they account for about 80% of all SSIs detected after any surgical procedure (4). SSIs following CS constitute a significant cause of morbidity and mortality, with risk of pain, delay in returning to normal activities, chronic pelvic pain, persistent seroma, depression, and impact on initiation or continuation of breastfeeding (3, 5). Moreover, SSIs after CS are associated with an increase in the duration of hospitalization, hospital costs, and community care costs following discharge (5). Therefore, it is essential to develop proper identification strategies for SSIs after CS to improve the quality of care and provide an effective surveillance system that can be used to assess the quality of SSIs control practice and increase awareness in both surgeons and patients, to reduce SSIs after CS rates (6, 7).

At present, there are no standard criteria for identifying SSIs after CS. The criteria defined by the Center for Disease Control and Prevention (CDC) are among the most used (9, 10). However, CDC criteria may present a degree of subjectivity since they include “diagnosis by attending physician” and “superficial incision that is deliberately opened by a surgeon, physician or physician designee” (8). Furthermore, it has been proven in other fields that CDC criteria have a poor agreement between attending surgeons in defining SSI (9). Since subjective definitions preclude comparability, there is the need for an accurate and objective system for SSIs identification, with a good inter-rater agreement, which can be adopted globally, and which also considers the post-discharge period since it is reported that about 79% of SSIs after CS occur post-hospital discharge (10, 11).

ASEPSIS score is an alternative and objective wound scoring system based on specific clinical findings that provide a quantitative analysis to determine the severity of the SSIs (12, 13). We hypothesized that the ASEPSIS score could overcome the impact of the subjectivity of CDC criteria. Still, its validity for SSIs after CS, also in terms of the inter-rater agreement, has yet to be evaluated. Therefore, we conducted this study to assess and compare the inter-rater agreement of the CDC criteria and the ASEPSIS score in identifying SSIs after CS.

## 2. Material and methods

A prospective observational study was performed on patients subjected to a CS at our institution (Clinica di Ostetricia e Ginecologia, Azienda Ospedaliero-Universitaria delle Marche, Università Politecnica delle Marche, Ancona, Italy). All patients were consecutively included from the start date of the study and excluded in case of age <18 years, stillbirth, unwillingness to participate, lack of availability for follow-up, or drop-out at follow-up. Women were managed according to standard care for the individual level of obstetric risk and any obstetric complications. Physicians of our institution provided indications for cesarean section according to routine clinical practice and local protocols. Patients were identified and approached for study inclusion after the decision to proceed with cesarean section, during the first postoperative day. On this occasion, all subjects received information about all study procedures and the processing of personal data. Informed consent was then requested, signed, and dated. According to local protocols, the following procedures are implemented in standard clinical practice for SSIs prevention after cesarean section: administration of pre-incision prophylactic antibiotic (cefazolin 2 gr IV and addition of azithromycin 500 mg IV in case of CS during labor or ruptured membranes), vaginal cleansing with 10% povidone-iodine, chlorhexidine-alcohol sponge for skin preparation, spontaneous placental removal, execution of subcuticular sutures if the subcutaneous fat thickness is >2 cm, and skin closure with intradermal suture (14). In the case of normal healing, postoperative wound care consisted of evaluation and cleaning on the third postoperative day (hospital discharge), performed by trained nurses without applying a new protective dressing. On this occasion, patients were also informed of the possibility of referral to our emergency room in case of wound complications after discharge. In the case of the onset of wound complications during hospitalization or after discharge, the wound was evaluated by the attending physician for the presence of serous exudate, purulent drainage from the superficial incision, localized pain or tenderness, localized swelling, erythema, heat, separation of deep tissues/spontaneous dehiscence or presence of an abscess. A wound swab specimen for culture was aseptically obtained in case of suspected bacterial infection. The attending physician evaluated whether to perform an incision to open the wound. At the end of any wound evaluation, the wound was cleaned, irrigated with saline solution, irrigated with povidone-iodine solution, and a protective dressing was applied. An antibiotic was administered according to clinical evaluation or positive culture results. The possible presence of fever (>38°C) was recorded. All possible adjunctive procedures (e.g., wound scarification, surgical drainage, or surgical closure) were performed according to the evaluation of the attending physician. The occurrence of other infectious complications (endometritis, extension to organ/space, sepsis) was recorded, and patients were treated based on the clinical situation, regardless of study participation. For the present study, in addition to the described care, the wound was photographed by trained nurses at each assessment with a dedicated digital camera, and the images were stored in digital archives not connected to the internet, accessible only to investigators. In the case of normal healing, the trained nurses evaluated and photographed the wound on the seventh and thirtieth postoperative day in an outpatient setting. The pictures included only the wound area and no identifying elements of the patient. All clinical data, such as whether the wound had been opened, the presence of drainage, and treatment with antibiotics, were collected and updated at each evaluation. Three expert surgeons reviewed the prospectively gathered data (complete with photographs) individually and independently. All clinical information was available for the raters (e.g., antibiotic prescription, adjunctive procedures, isolation of bacteria from the wound). The three expert surgeons were selected among those working at our structure, with at least ten years of experience and five hundred cesarean sections performed, with follow-up of cesarean wounds. Since all identifying information was removed, the reviewers were completely blinded. Reviewers classified each wound with the use of CDC criteria for SSIs. Reviewers also assigned an ASEPSIS score to each wound assessment to generate a cumulative 30-day ASEPSIS score.

### 2.1. CDC criteria and ASEPSIS score

CDC criteria for identifying SSIs were applied by the reviewers to each wound according to CDC definitions (8) and categorized as “no SSI”, “superficial incisional SSI”, or “deep incisional SSI”. The ASEPSIS score was calculated by assigning a score based on the presence of erythema, serous exudate, purulent exudate, and deep-tissue separation according to the percentage of the wound affected by each process (12, 13). Additional points were awarded for antibiotic treatment, incision and drainage, isolation of bacteria from the wound, and inpatient stay. A score greater than 20 defined the presence of an SSI. Scores were grouped into the following categories: satisfactory healing (0–10), disturbance of healing (11–20), minor SSI (21–30), moderate SSI (31–40), and severe SSI (>40).

### 2.2. Variables and outcomes

The following background and clinical variables were collected: age, BMI, weight gain during pregnancy (kg), tobacco use during pregnancy, number of previous pregnancies, parity (number of previous births with a gestational age ≥24 weeks), diagnosis of diabetes during pregnancy (gestational diabetes or pre-existing diabetes), diagnosis of hypertensive disorders of pregnancy, colonization of Group B streptococcus (GBS) at the recto-vaginal swab, gestational weeks at CS, and duration of hospitalization (days). If the CS was performed during labor, we reported the characteristics of the amniotic fluid (clear or meconium-stained), the number of vaginal examinations during labor, the premature rupture of membranes >18 h, the duration of labor (hours), the cervical dilatation (cm), and the presence of fever (>38°C) during labor. The American Society of Anesthesiologists (ASA) physical status classification, the type of anesthesia (regional or general), the duration of surgery (min), the type of skin incision (Pfannenstiel or longitudinal), the time between skin disinfection and incision (min), the thickness of the subcutaneous tissue (cm), the suture length (cm), the intraoperative blood loss (ml), the pre-operative and postoperative Hb values (g/dl), and pre-operative white blood cell (WBC) count (/mmc) were recorded. The local ethical committee (Comitato Etico Regionale Marche) approval was obtained before the start of the study (Prot. 2021/77). The primary outcome was the inter-rater agreement between the three surgeons in assessing SSIs after CS, using CDC definitions and the ASEPSIS score.

### 2.3. Statistical analysis

Statistical software SPSS 27.0 (SPSS Inc., Chicago, IL, USA) was used. The normality of each variable was evaluated with the D'Agostino–Pearson test. Normally distributed variables were expressed as arithmetic mean ± standard deviation (SD), while Not-normally distributed variables were reported as median and interquartile range (IQR). Qualitative variables were expressed as numbers and percentages. Chi-squared test, Fisher's exact test, Mann-Whitney test, or *t*-test were used for variable comparison, as appropriate. Krippendorff's Alpha with linear weights was run to determine the agreement between three surgeons about CDC definitions and ASEPSIS score (15). A *p*-value <0.05 was regarded as statistically significant. The Krippendorff's Alpha values and confidence intervals obtained from CDC definitions and ASEPSIS were compared to test any difference between them; no or minimal overlap between the confidence intervals was considered indicative of a statistically significant difference.

### 2.4. Number of subjects and sample size

The kappaSize v1.2 package for R Software© (R Foundation for Statistical Computing, 2016) was used to determine the required sample size with a confidence interval-based approach for inter-rater agreement of three raters for three outcome categories (16). The kappaSize v1.2 package was chosen in the absence of a specific method for sample size determination for Krippendorff *α*, also considering that Krippendorff *α* is a generalization of several agreement indices, including kappa (17). For sample size determination, categories from ASEPSIS were grouped as follows: satisfactory healing and disturbance of healing as no SSI, minor and moderate SSI, and severe SSI (score >40). The reported prevalence of SSI after CS is 9.0% for superficial SSI (minor and moderate SSI for ASEPSIS) and 1.0% for deep SSI (severe SSI for ASEPSIS) (18, 19). Previous literature reports a weighted kappa of 0.5 for inter-rater agreement in assessing SSI with CDC definitions (13) and 0.9 with ASEPSIS score (13, 19). We estimated the sample size for the present study with an alpha of 0.05, a 95% confidence interval, a precision level set to 0.2 on each side, and a maximum error of 0.2. Consequently, the lower limit of the confidence interval for CDC definitions was 0.3, and the upper limit was 0.7. The minimum sample size resulted in 102 subjects for evaluation of inter-rater agreement for CDC definitions. For ASEPSIS, the lower limit of the confidence interval was 0.7, and the upper limit was 0.99. The minimum sample size resulted in 80 subjects for evaluation of inter-rater agreement for ASEPSIS score. Considering that the reviewers evaluated the included patients with CDC definitions and ASEPSIS score, the minimum sample size was 102 subjects. Assuming a dropout rate of 20%, we aimed to include at baseline at least 128 patients.

## 3. Results

Patients subjected to a CS at our institution were prospectively included from June 2021 to December 2021. In this period, 132 patients who fulfilled the inclusion and exclusion criteria were included at baseline. Among them, 22 (16.7%) patients did not attend all the follow-up visits and were excluded. Thus, 110 patients were included in the final analysis. Table 1 reports the demographics of the study population. Krippendorff's Alpha with linear weights was run to determine the extent of agreement among three expert surgeons on identifying SSI after CS, both with CDC criteria and with ASEPSIS score. The distribution of subjects by rater and category of CDC criteria and ASEPSIS score is reported in Table 2. Among raters, the prevalence of deep SSI, superficial SSI, and no SSI ranged from 1.8% to 3.6%, from 10.0% to 14.6%, and from 81.8% to 88.2%, respectively. The weighted *α* coefficient for CDC criteria was 0.587 (standard error 0.089, 95% CI, 0.411–0.763), *p* < 0.001. According to Altman's interpretation of the weighted agreement coefficient, *α* for CDC criteria resulted in a “moderate” agreement. The prevalence range among raters of severe SSI, moderate/minor SSI, and no SSI was 0.9%–2.7%, 1.8%–3.6%, and 94.6%–97.3%, respectively (Table 2). The weighted *α* coefficient for the ASEPSIS score was 0.856 (standard error 0.062, 95% CI, 0.733–0.980), *p* < 0.001, which corresponds to a “very good” agreement according to Altman's interpretation of the weighted agreement coefficient. There was minimal overlap between the confidence intervals from CDC criteria and the ASEPSIS score. Table 3 compares SSI risk factors between patients with an SSI identified by at least one rater (*n* = 28) and patients without SSI identification (*n* = 82). Patients in whom the CS was performed during labor presented a higher risk of SSI (32.1% vs. 11.0%) (Table 3). Supplementary Figure S1 compares all cases with an SSI identified by at least one rater in terms of CDC criteria or ASEPSIS score class of SSI. No other infectious complication (endometritis, extension to organ/space, sepsis) was recorded among the included patients.

**Table 1:** Demographic and perinatal characteristics of the study population (*n* = 110).

Variable	Study population (*n* = 110)
Maternal age at delivery (years)	34.8 ± 5.5
Maternal BMI	27.6 ± 5.6
Weight gain (kg)	11 (8–14)
Tobacco use during pregnancy	10 (9.1%)
Number of previous pregnancies	1 (0–2)
Parity	1 (0–1)
Nulliparous	50 (45.5%)
Gestational diabetes	29 (26.4%)
Hypertensive disorders of pregnancy	13 (11.8%)
Gestational age at cesarean section (weeks)	37.9 ± 2.9
ASA physical status classification	1 (1–1)

Data are reported as *n* (%), median (IQR), or mean ± SD, as appropriate.

**Table 2:** Distribution of subjects by rater and category of CDC criteria and ASEPSIS score (*n* = 110).

CDC criteria	No SSI	Superficial SSI	Deep SSI	Total
Rater 1	90 (81.8%)	16 (14.6%)	4 (3.6%)	110
Rater 2	97 (88.2%)	11 (10.0%)	2 (1.8%)	110
Rater 3	95 (86.4%)	11 (10.0%)	4 (3.6%)	110
ASEPSIS score	No SSI	Moderate/Minor SSI	Severe SSI	Total
Rater 1	99 (94.6%)	3 (2.7%)	3 (2.7%)	110
Rater 2	107 (97.3%)	2 (1.8%%)	1 (0.9%)	110
Rater 3	105 (95.5%)	4 (3.6%%)	1 (0.9%)	110

Data are reported as *n* (%).

SSI, surgical site infection; No SSI, ASEPSIS score 0–20; Moderate/minor SSI, ASEPSIS score 21–40; Severe SSI, ASEPSIS score >40.

**Table 3:** Comparison of risk factors for SSIs between patients with an SSI identified by at least one rater (*n* = 28) and patients without SSIs identification (*n* = 82).

Variable	SSIs (*n* = 28)	No SSIs (*n* = 82)	*p*
Maternal age at delivery (years)	35.9 ± 4.7	34.4 ± 5.8	0.2192
Maternal BMI	26.2 ± 5.5	28.1 ± 5.5	0.1174
Weight gain (kg)	11 (8–14)	11 (9–14)	0.8710
Tobacco use during pregnancy	5 (17.9%)	5 (6.1%)	0.0621
Number of previous pregnancies	1 (0–2)	1 (0–2)	0.3449
Gestational diabetes	8 (28.6%)	21 (25.6%)	0.7568
Hypertensive disorders of pregnancy	3 (10.7%)	10 (12.2%)	0.8326
Gestational age at cesarean section (weeks)	37.9 ± 3.1	37.9 ± 2.8	1.0000
Group B streptococcus colonization	2 (7.1%)	5 (6.1%)	0.8521
Duration of hospitalization (days)	5 (4–9)	4 (4–5)	0.5671
ASA score	1 (1–1)	1 (1–1)	0.6501
Regional anesthesia	27 (96.4%)	80 (97.6%)	0.7366
Duration of surgery (min)	47 (40–60)	45 (35–52)	0.1035
Pfannenstiel incision	28 (100%)	81 (98.8%)	0.5622
Time disinfection-incision (min)	2 (2–3)	2 (2–3)	0.1353
Thickness of subcutaneous tissue (cm)	2 (2–3)	2 (2–3)	0.6475
Subcuticular tissue suture	12 (42.9%)	33 (40.2%)	0.8028
Suture length (cm)	15 (14–15)	15 (14–15)	0.8812
Intraoperative blood loss (ml)	388 (200–590)	400 (238–615)	0.2076
Pre-operative Hb (g/dl)	11.9 ± 1.3	11.7 ± 1.1	0.4299
Post-operative Hb (g/dl)	11.1 ± 1.2	10.8 ± 1.2	0.2559
Pre-operative WBC count (/mmc)	8,606 (6,925–10,114)	8,715 (7,925–10,073)	0.5301
CS during labor	9 (32.1%)	9 (11.0%)	0.0095
Meconium-stained amniotic fluid^a^	2 (22.2%)	1 (11.1%)	1.0000
Number of vaginal examinations^a^	3 (5–7)	7 (3–10)	0.3979
Premature rupture of membranes >18 h^a^	3 (33.3%)	4 (44.4%)	1.0000
Duration of labor (hours)^a^	18 (10–25)	13 (9–20)	0.4006
Cervical dilatation (cm)^a^	7 (4–10)	8 (3–9)	0.7190
Fever (>38°C) during labor^a^	2 (22.2%)	0 (0.0%)	0.4706

Data are reported as *n* (%), median (IQR), or mean ± SD, as appropriate.

As appropriate, the chi-squared test, Fisher's exact test, Mann–Whitney test, or *t*-test were used for comparison.

^a^ These data refer to the 18 patients who underwent CS during labor (nine with SSIs and nine without SSIs).

## 4. Discussion

Results from the present study showed that the ASEPSIS score had a “very good” agreement between raters for SSIs identification after CS (*α* = 0.856, 95% CI, 0.733–0.980, *p* < 0.001). On the other hand, the inter-rater agreement of CDC criteria resulted as “moderate” (*α* = 0.587, 95% CI, 0.411–0.763, *p* < 0.001). Therefore, considering the minimal overlap between the *α* confidence intervals of the two methods, the ASEPSIS score seems to be a more objective method in identifying SSIs after CS. In other words, there were fewer discrepancies among operators evaluating CS surgical wounds. This study was the first to report the inter-rater agreement of ASEPSIS score for SSIs after CS. Data regarding the inter-rater agreement of ASEPSIS score are available from other fields of surgery and are in line with our results. Hedrick et al. reported in 2015 about the agreement between CDC criteria and ASEPSIS score in identifying SSI after elective colorectal surgery, showing that ASEPSIS score yielded reliable measures for comparison (*κ* = 0.83) and that CDC definitions had a relatively poor agreement (*κ* = 0.55) (13). Regarding orthopedic wound infections, the inter-rater agreement of the ASEPSIS score was 90% in the study by Copanitsanou et al. of 2019 (20). In general surgery, Byrne et al. reported an inter-rater agreement of 0.96 for the ASEPSIS score (21). ASEPSIS score has also been effective in predicting clinical outcomes: the study from Campwala et al. of 2019 reported that ASEPSIS score effectively predicted implant-based breast reconstruction failure (22). Hasselmann et al. reported in 2019 that the ASEPSIS score may be useful in evaluating the effectiveness of negative pressure wound therapy on closed incisions after inguinal vascular surgery (23). The finding of a higher risk of SSI in patients in whom the CS was performed during labor was expected since this condition was previously reported in the literature as a risk factor for SSIs after CS (24, 25). Analyzing the distribution of cases with an SSI identified by at least one reviewer, we noticed that most of the superficial SSIs identified by CDC criteria resulted in “no SSI” according to the ASEPSIS score (Supplementary Figure S1). Similar findings were observed by Wilson et al. in 2004 (6); in their study conducted on multiple surgical subspecialties, CDC criteria diagnosed greater infection severity than the ASEPSIS score. Indeed, 42% of wounds classified as “disturbance of healing” from ASEPSIS were classified as infected by the CDC criteria, with a moderate inter-method agreement (*κ* = 0.43, 95% CI, 0.40–0.46) (6). The CDC category of superficial SSI presented poor agreement between the two methods (*κ* = 0.11, 95% CI, 1.0–0.23), also in the study by Henriksen et al. of 2010, conducted on patients subjected to abdominal surgery (26). The clinical significance of this difference between the two methods cannot be clearly defined. CDC criteria may identify a higher rate of SSIs after CS, which may not be clinically relevant, or the ASEPSIS score may miss some potentially relevant SSIs with a delay in diagnosis and treatment (27, 28). Currently, in the absence of a standard reference method to define clinically relevant SSIs after CS, it is not possible to discriminate whether all identified SSIs by CDC criteria or ASEPSIS score may potentially have consequences for patients. However, the ASEPSIS score seems to be a more objective and reliable method to design future studies to evaluate its association with the clinical significance of SSIs. Regarding SSIs surveillance after CS, we believe the ASEPSIS score could provide a more objective view since it seems less influenced by minor infections with presumably little effects on the healthcare system. The limitations of this study are related to the inclusion of data from a single institution and the fact that the reviewers did not directly manage the included patients with SSIs. Moreover, our analysis focused on CS, so the results are not immediately generalized to other surgical interventions. However, the results are strengthened by having included a large number of patients with an “a priori” sample size determination method to obtain an adequate power of the study and by having reported for the first time about the inter-rater agreement of ASEPSIS score for SSIs after CS by blinded review of prospectively collected photographic data. Totty et al. reported in 2018 about the good correlation between face-to-face clinical and remote photographic review with ASEPSIS score, but in the case of vascular surgery (29). Therefore, we have proved how photographic assessment may be a reliable CS wound evaluation method. Using photographic data could improve surveillance and care of SSIs by implementing a mobile application-based system. Indeed, developing a mobile application allowing the patient to take a picture of her CS wound and send it directly to healthcare professionals will represent an effective and low-cost tool for SSIs care and surveillance. Healthcare professionals would directly categorize the wound as infected or not using the ASEPSIS score, and deep-learning techniques could help them. This mobile application-based system would achieve the requirements indicated by Sawyer et al. in 2019 for the implementation of digital imaging in clinical management and surveillance of SSIs: it will allow identification of SSIs regardless of the patient's care site and post-hospital discharge, will allow a more rapid and accurate diagnosis, will decrease the frequency with which patients present to healthcare facilities with surgical site concerns, and it will be able to provide a and would make possible a more rapid data collection for surveillance (28). Implementing a mobile application-based system would also overcome the reported complexity of calculation and use of the ASEPSIS score in daily practice (7, 13). Prior experience with mobile applications for SSIs monitoring has been reported with promising results, but the possibility of acquiring images has never been implemented (30, 31). Future validation studies are needed to evaluate the effectiveness of a new-developed mobile application-based system to identify SSIs after CS. In conclusion, the ASEPSIS score presents a very good inter-rater agreement for SSIs identification after CS, resulting in a more objective method than CDC criteria, which presented a moderate inter-rater agreement. ASEPSIS score could represent a helpful tool for clinical management and surveillance of SSIs after CS. Dedicated studies should further investigate its usefulness in a mobile application-based system.

## Data Availability

The raw data supporting the conclusions of this article will be made available by the authors, without undue reservation.

## Supplementary material

Supplementary Figure 1 Comparison of all cases with an SSI identified by at least one rater in terms of CDC criteria or ASEPSIS score class.
