# Supplementary figures and images for: Inter-rater agreement of CDC criteria and ASEPSIS score in assessing surgical site infections after cesarean section: a prospective observational study
Source: Front Surg. 2023 Aug 22;10:1123193. doi: 10.3389/fsurg.2023.1123193 (PMC10477579; doi:10.3389/fsurg.2023.1123193)

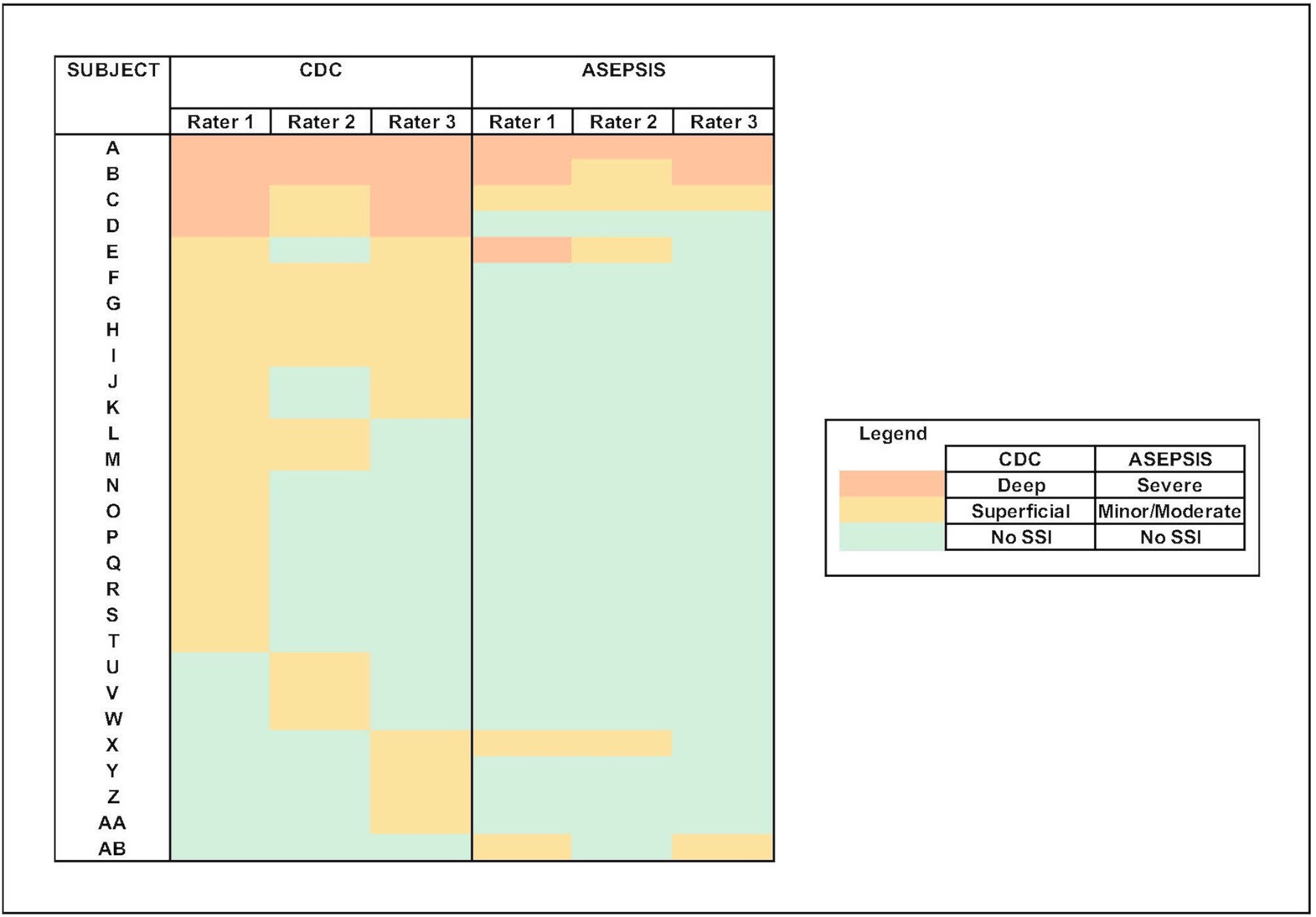

Supplement: Supplementary Figure 1 — Comparison of all cases with an SSI identified by at least one rater in terms of CDC criteria or ASEPSIS score class. [file Image1.jpeg]
